# 32种氧化型染料的高效液相色谱定量及高效液相色谱-串联质谱确证方法

**DOI:** 10.3724/SP.J.1123.2022.03003

**Published:** 2022-09-08

**Authors:** Zhiming ZHOU, Jing LI, Zhanghao CHEN, Yingying WU, Tuliu LU, Shuxiong XIAO

**Affiliations:** 国家药品监督管理局化妆品风险评估重点实验室(广东省药品检验所), 广东 广州 510525; National Medical Products Administration Key Laboratory for Safety Risk Assesment of Cosmetics (Guangdong Institute for Drug Control), Guangzhou 510525, China

**Keywords:** 高效液相色谱, 高效液相色谱-串联质谱, 氧化型染料, 染发类产品, high performance liquid chromatography (HPLC), high performance liquid chromatography-tandem mass spectrometry (HPLC-MS/MS), oxidative dyes, hair dye products

## Abstract

染发类产品中氧化型染料种类多,实际样品测定时干扰多,建立染发类产品中多种常用染料的测定方法,为该类产品的有效监管提供技术手段十分必要。该研究根据染料使用频率分组,采用能够屏蔽硅羟基和金属离子影响的C_18_柱,优化了《化妆品安全技术规范》(2015年版)中32种染料的高效液相色谱法(HPLC)并建立了高效液相色谱-串联质谱(HPLC-MS/MS)确证方法。样品以10 g/L亚硫酸氢钠水溶液为抗氧化剂,用无水乙醇-水(1∶1, v/v)混合溶液冰浴超声提取10 min。HPLC方法采用甲醇、乙腈和磷酸盐缓冲液为流动相分两个液相色谱条件进行梯度洗脱分离,于280 nm波长下检测,其中一个HPLC条件中的相互干扰组分均在另一个HPLC条件下完全分离,避免了实际样品检测时组分间的干扰,并排除了32种以外的其他15种常用染料的干扰。HPLC-MS/MS方法分别采用5 mmol/L乙酸铵水溶液-乙腈和5 mmol/L乙酸水溶液-乙腈为正离子和负离子模式下的流动相,电喷雾离子模式下用多反应监测(MRM)模式进行定性和定量分析。HPLC和HPLC-MS/MS两个方法中,日内精密度和48 h内稳定性的相对标准偏差(RSD)<10%,回收率为82.6%~114.9%(RSD<10%)。HPLC方法中32种染料在大约10~500 mg/L范围内线性关系良好(*r*^2^>0.99),检出限为9.7~40.1 μg/g; HPLC-MS/MS方法中氢醌线性范围为2.0~79.7 mg/L,检出限为8.0 μg/g,其他组分线性范围约为0.1~4 mg/L,检出限为0.01~0.4 μg/g。采用HPLC、HPLC-MS/MS两个方法和《化妆品安全技术规范》方法同时测定实际样品,共检出16种染料,检出含量范围为58~25160 μg/g。3个方法检测结果的RSD为1.9%~10.1%。该研究增加了HPLC-MS/MS确证方法,适应化妆品法定检验中的未知物确认程序;方法简便快速,结果准确,专属性强,具有较好的通用性和可操作性。

## Abstract (en)

Various types of oxidative dyes used in hair dye products possess poor stability and have varying frequency of use. Interference problems also frequently arise in actual sample measurements. Therefore, it is necessary to establish a simple, rapid, accurate, and specific method for the determination of common dyes in hair dye products for their effective regulation. In this study, dyes were grouped according to their frequency of use. Using a C_18_ column that minimizes the silanol effect and influence of metals, the quantitative high performance liquid chromatography (HPLC) method for 32 dyes listed in Safety and Technical Standards for Cosmetics (2015 edition) was optimized, and a high performance liquid chromatography-tandem mass spectrometry (HPLC-MS/MS) confirmatory method for the dyes was established. The samples were extracted using a mixed solution of ethanol-water (1∶1, v/v) with 10 g/L sodium bisulfite solution as an antioxidant, vortexed and mixed, and then extracted by ultrasonication in an ice bath for 10 min. Methanol, acetonitrile, and phosphate buffer were used as the mobile phases in the HPLC analysis. Additionally, two different elution conditions (chromatographic gradient) were used for the separation of 32 oxidative dyes, which were detected at a wavelength of 280 nm. The HPLC separations were compared using columns of particle sizes 5 μm and 2.7 μm; 5 μm C_18_ columns with better anti-interference and antiblocking ability were selected. Satisfactory separation was achieved for all three commercial C_18_ columns with a particle size of 5 μm, and the method had good general usability. In condition 1, 17 commonly used dyes and three less commonly used dyes were assigned to group Ⅰ and separated by HPLC; in condition 2, eight banned dyes and four other less commonly used dyes were assigned to group Ⅱ and separated by HPLC. The HPLC-MS/MS method used 5 mmol/L ammonium acetate aqueous solution-acetonitrile and 5 mmol/L acetic acid aqueous solution-acetonitrile as mobile phases in the positive and negative ion modes, respectively. Multiple reaction monitoring (MRM) was performed for qualitative and quantitative analyses in the electrospray ionization mode. Under the examined conditions, six pairs of isomers were well resolved. For the HPLC and HPLC-MS/MS methods, the relative standard deviations (RSDs) of the intra-day precision and 48 h stability tests were less than 10%. The recoveries were between 82.6% and 114.9% (RSD<10%). In the HPLC method, 32 dyes showed good linearity in an approximate range of 10-500 mg/L (*r*^2^>0.99), and the limits of detection (LODs) were 9.7-40.1 μg/g. The linear range of hydroquinone in the HPLC-MS/MS method was 2.0-79.7 mg/L, and the LOD was 8.0 μg/g; the linear ranges of the other components were approximately 0.1-4 mg/L, and the LODs were 0.01-0.4 μg/g. The actual samples were simultaneously measured by HPLC, HPLC-MS/MS, and the standard method. Finally, 16 of the 32 dyes were detected, and the detected contents ranged from 58 to 25160 μg/g. The RSDs of the results obtained from the three detection methods were between 1.9% and 10.1%. All detected components were within the limits of group Ⅰ of this method. In comparison with methods reported in the literature and the standard method, this method covers all components for the routine regulatory inspection of oxidative dyes in cosmetics. The method can separate the commonly used dyes under the same HPLC conditions and avoid interference from 15 other commonly used dyes during the analysis of actual samples. A suitable HPLC-MS/MS confirmatory method was also established for the identification of currently unknown substances in the statutory inspection of cosmetics. The method is simple, rapid, accurate, and specific with general usability and good operability.

氧化型染料主要为苯胺类及苯酚类化合物,这些化合物有着不同程度的致敏和致畸性^[1]^,在化妆品中使用时需严格控制,这一直是监管的重点^[2]^。氧化型染料的准确定量是分析检测的难点,一是由于染发膏膏体黏稠、基体溶散困难,溶散不充分会影响提取率;二是氧化型染料光、热稳定性差,样品处理及分析时间过长时组分可能降解,测得含量偏低;三是染料组分多、使用浓度跨度大,检测时各组分间往往存在相互干扰且可能同时需要进行常量和痕量测定,并要求检验结果与配方、标签比对;四是苯胺类碱性物质在流动相中易离子化,色谱保留弱,固定相的键合方式及流动相的酸碱度对峰形和保留影响明显。所以针对氧化型染料,开发操作简便、定性定量准确、适用不同应用场景和检测需求的检测方法具有现实意义。

大部分染料挥发性弱,气相色谱和气相色谱-串联质谱方法应用受限^[3,4]^,标准及文献报道中氧化型染料的测定以高效液相色谱(HPLC)^[5,6]^和高效液相色谱-串联质谱(HPLC-MS/MS)法^[7,8]^为主;样品中不同染料含量差异大,同时进行常量及痕量检测易造成离子源污染,不适于采用HPLC-MS/MS直接测定,一般采用HPLC定量检测、HPLC-MS/MS确证的解决方案。《化妆品安全技术规范》(2015年版)(以下简称《规范》)^[9]^收录了75种准用染料及两种液相色谱法:“7.1对苯二胺等8种组分”涵盖组分少,已不能满足监督检验的需求;“7.2对苯二胺等32种组分”覆盖种类多、定量准确,但需要分3个HPLC系统;采用的C_16_柱和离子对试剂耐用性欠佳;样品测定时存在干扰;缺乏质谱确证方法,未能满足监督检验未知物确认程序需要。严巍等^[10]^采用C_18_柱,分3个HPLC系统,根据p*K*_a_值调整流动相及pH值的方式改善了峰形,但仍不能避免样品的干扰问题。左雪等^[11,12]^建立了单一HPLC系统分别分离33种和40种染料,避免了样品检测中的干扰,但只覆盖了《规范》中法定检测的32种染料中的27种和29种常用成分,不能涵盖目前监督检验要求的所有组分。

本研究优化了《规范》中法定检测的32种氧化型染料的HPLC定量测定方法,并建立了HPLC-MS/MS确证方法。HPLC法将17种常用染料及3种较少用染料共20种组分作为Ⅰ组在HPLC条件1下完成分离,8种禁用染料及4种较少用染料共12种组分作为Ⅱ组在HPLC条件2下分离,其中一个HPLC条件中的相互干扰组分均在另一个HPLC条件下完全分离,避免了实际样品检测时组分间的干扰,并排除了32种以外的其他15种常用染料的干扰。在日常检测时,建议可优先选择HPLC法进行含量测定;当出现相同保留时间下光谱吸收匹配存疑、检出结果与标签/注册资料不符时,可采用HPLC-MS/MS法进一步定性确证;当样品某组分含量低于HPLC法的定量浓度、存在杂质峰干扰导致HPLC法无法准确定量时,可采用HPLC-MS/MS法定量确证。方法准确可靠,专属性强,具有较好的适用性和通用性。

## 1 实验部分

### 1.1 仪器、材料与试剂

SHIMADZU LC-20AT液相色谱仪(配二极管阵列检测器); SCIEX Triple Quad 5500液相色谱-质谱联用仪;IkaLabDancer型涡旋混合器;Millipore Milli Q Reference型超纯水发生器;Elma S300H超声波清洗器;Mettler MSDU电子天平(十万分之一)。

*p*-苯二胺、*p*-氨基苯酚、*o*-苯二胺、*o*-氨基苯酚、2-硝基-*p*-苯二胺、甲苯-3,4-二胺、苯基甲基吡唑啉酮、4-氨基-3-硝基苯酚、2-氨基-3-羟基吡啶、*p*-甲基氨基苯酚硫酸盐、2,6-二氨基吡啶、*N*,*N*-二乙基-*p*-苯二胺硫酸盐、1-萘酚均购自美国Sigma Aldrich公司;甲苯-2,5-二胺硫酸盐、*m*-氨基苯酚、2-氯-*p*-苯二胺硫酸盐、间苯二酚、4-氨基-2-羟基甲苯、2-甲基雷琐辛、6-氨基-*m*-甲酚、*N*,*N*-二乙基甲苯-2,5-二胺盐酸盐、*m*-苯二胺、氢醌、4-硝基-*o*-苯二胺、4-氯雷琐辛、2,7-萘二酚、*N*-苯基-*p*-苯二胺、1,5-萘二酚均购自梯希爱(上海)化成工业发展有限公司(TCI); 2,4-二氨基苯氧基乙醇盐酸盐、*N*,*N*-双(2-羟乙基)-*p*-苯二胺硫酸盐、6-羟基吲哚均购自上海麦克林生化科技有限公司(MACKLIN)。所有对照品纯度均大于96%。

甲醇、乙腈均为色谱纯,购自霍尼韦尔(中国)有限公司;磷酸氢二钾、无水乙醇、亚硫酸氢钠、磷酸均为分析纯,购自广州化学试剂厂。

磷酸盐缓冲液:称取三水合磷酸氢二钾4.56 g,加水1 L溶解,混匀,制成20 mmol/L的磷酸盐缓冲液,用磷酸溶液调节pH至7.5,经0.45 μm微孔滤膜过滤。

样品为市售氧化型染发产品,含染发膏和氧化乳,取染发膏供含量测定。

### 1.2 标准溶液的配制

#### 1.2.1 单标准储备溶液的配制

称取各染料对照品0.1 g(精确到0.0001 g),分别置于10 mL容量瓶中。由于溶解性差异,甲苯-2,5-二胺硫酸盐、2-氯-*p*-苯二胺硫酸盐用2 g/L亚硫酸氢钠水溶液溶解定容;甲苯-3,4-二胺用2 g/L亚硫酸氢钠水溶液和甲醇(1∶9, v/v)溶解定容;2-硝基-*p*-苯二胺和4-硝基-*o*-苯二胺将称样量减至25 mg(精确到0.00001 g),用2 g/L亚硫酸氢钠水溶液-甲醇(1∶9, v/v)溶解定容,配制成质量浓度约2.5 g/L的溶液。其余27种染料以2 g/L亚硫酸氢钠水溶液-甲醇(1∶1, v/v)溶解定容,配制成质量浓度约为10 g/L的各染料单标准储备溶液。

单标准储备溶液保存于0~4 ℃冰箱中,48 h内使用。

#### 1.2.2 混合标准溶液的配制

32种染料混合标准溶液:取32种染料的单标准储备溶液适量于10 mL容量瓶中,用2 g/L亚硫酸氢钠水溶液-甲醇(1∶1, v/v)稀释至刻度,配制成质量浓度为250 mg/L的32种染料混合标准溶液。

Ⅰ组混合标准溶液:取*p*-苯二胺、*p*-氨基苯酚、甲苯-2,5-二胺硫酸盐、*m*-氨基苯酚、2-氯-*p*-苯二胺硫酸盐、间苯二酚、2-硝基-*p*-苯二胺、4-氨基-2-羟基甲苯、2-甲基雷琐辛、苯基甲基吡唑啉酮、4-氨基-3-硝基苯酚、2,4-二氨基苯氧基乙醇盐酸盐、4-氨基-*m*-甲酚、2-氨基-3-羟基吡啶、*N*,*N*-双(2-羟乙基)-*p*-苯二胺硫酸盐、2,6-二氨基吡啶、6-羟基吲哚、4-氯雷琐辛、1,5-萘二酚、1-萘酚的单标准储备溶液适量于10 mL容量瓶中,用2 g/L亚硫酸氢钠水溶液-甲醇(1∶1, v/v)稀释至刻度,配制成质量浓度为500 mg/L的Ⅰ组混合标准溶液。

Ⅱ组混合标准溶液:取*o*-苯二胺、*o*-氨基苯酚、甲苯-3,4-二胺、6-氨基-*m*-甲酚、*N*,*N*-二乙基甲苯-2,5-二胺盐酸盐、*m*-苯二胺、氢醌、*p*-甲基氨基苯酚硫酸盐、4-硝基-*o*-苯二胺、*N*,*N*-二乙基-*p*-苯二胺硫酸盐、2,7-萘二酚、*N*-苯基-*p*-苯二胺的单标准储备溶液适量于10 mL容量瓶中,用2 g/L亚硫酸氢钠水溶液-甲醇(1∶1, v/v)稀释至刻度,配制成质量浓度为500 mg/L的Ⅱ组混合标准溶液。

#### 1.2.3 混合标准系列溶液的配制

分别取Ⅰ组和Ⅱ组混合标准溶液适量,用2 g/L亚硫酸氢钠水溶液-甲醇(1∶1, v/v)稀释,配制成质量浓度为10、25、50、100、250、500 mg/L的Ⅰ组和Ⅱ组混合标准系列溶液。混合标准系列溶液临用现配。

### 1.3 样品处理

称取样品0.5 g(精确至0.001 g)于25 mL具塞比色管中,加入10 g/L亚硫酸氢钠水溶液2.0 mL,用无水乙醇-水(1∶1, v/v)混合溶液溶解并定容,涡旋振荡30 s使试样与提取溶剂充分混匀,冰浴超声提取10 min,如样品浑浊可在10000 r/min下离心5 min。取上清液经0.45 μm滤膜过滤,滤液作为待测溶液,并尽快测定。

### 1.4 仪器条件

#### 1.4.1 HPLC条件

色谱柱:Shimadzu Shim-pack GIST C_18_柱(250 mm×4.6 mm, 5 μm);柱温:25 ℃;进样体积:5 μL;流速:1.0 mL/min;检测波长:280 nm。分两个HPLC条件(条件1、条件2)分离,流动相及梯度洗脱条件见表1。

**表 1:** 32种染料的HPLC及HPLC-MS/MS流动相梯度条件

HPLC condition 1					HPLC condition 2				HPLC-MS/MS condition			
Time/min	φ(A)/%	φ(B)/%	φ(C)/%		Time/min	φ(A)/%	φ(B)/%		Time/min	φ(D)/%		φ(C)/%
0	96	2	2		0	92	8		0	95		5
15	96	2	2		8	92	8		4	95		5
22	90	5	5		35	50	50		11	80		20
35	50	10	40		45	50	50		16	30		70
50	50	10	40		45.1	92	8		17	5		95
50.1	96	2	2		50	92	8		19	5		95
55	96	2	2						19.1	95		5

A: 20 mmol/L dipotassium hydrogen phosphate (pH 7.5 adjusted by phosphoric acid); B: methanol; C: acetonitrile; D: acetonitrile-5 mmol/L ammonium acetate aqueous solution (positive ion mode) or acetonitrile-5 mmol/L acetic acid aqueous solution (negative ion mode).

#### 1.4.2 HPLC-MS/MS条件

色谱柱:Waters atlant T_3_ (150 mm×2.1 mm, 3 μm);柱温:30 ℃;进样体积:2 μL;流速:0.3 mL/min;流动相及梯度洗脱条件见表1。离子源:电喷雾离子(ESI)源;监测模式:多反应监测模式;监测离子对及相关参数设定见表2;雾化气流速:3 L/min;干燥气流速:15 L/min;脱溶剂管温度:250 ℃;离子源加热温度:400 ℃;碰撞气:Ar, 230 kPa。

**表 2:** 32种染料的电离模式、母离子、子离子、碰撞能及保留时间

Dye No.	Dye	Ionization mode	Parent ion (m/z)	Daughter ion (m/z) (collision energy/eV)	t_R_/min
1	p-phenylenediamine	+	109.2	65.2 (31)	2.7
	(p-苯二胺)			92.1 (26)	
2	p-aminophenol	+	110.0	65.1 (28)	3.7
	(p-氨基苯酚)			93.0 (38)	
3	toluene-2,5-diamine sulfate	+	123.1	108.2 (24)	9.9
	(甲苯-2,5-二胺硫酸盐)			77.1 (40)	
4	m-aminophenol	+	109.9	65.1 (28)	6.3
	(m-氨基苯酚)			92.9 (22)	
5	o-phenylenediamine	+	109.0	65.2 (33)	8.0
	(o-苯二胺)			92.1 (22)	
6	2-chloro-p-phenylenediamine sulfate	+	143.1	108.1 (25)	9.6
	(2-氯-p-苯二胺硫酸盐)			80.1 (36)	
7	o-aminophenol	+	110.0	65.1 (30)	9.1
	(o-氨基苯酚)			92.1 (22)	
8	resorcinol	-	109.1	65.0 (17)	8.9
	(间苯二酚)			41.0 (23)	
9	2-nitro-p-phenylenediamine	+	154.0	119.1 (22)	11.0
	(2-硝基-p-苯二胺)			135.8 (13)	
10	toluene-3,4-diamine	+	123.0	77.1 (37)	13.6
	(甲苯-3,4-二胺)			106.0 (22)	
11	4-amino-2-hydroxytoluene	+	124.0	109.0 (26)	13.2
	(4-氨基-2-羟基甲苯)			77.1 (35)	
12	2-methylresorcinol	-	123.0	55.0 (34)	10.6
	(2-甲基雷琐辛)			79.0 (17)	
13	6-amino-m-cresol	+	124.1	106.0 (30)	14.2
	(6-氨基-m-甲酚)			77.1 (36)	
14	pyrazolone methyl pyrazolone	+	175.2	133.0 (26)	14.7
	(苯基甲基吡唑啉酮)			65.0 (46)	
15	N,N-diethyltoluene-2,5-diamine hydrochloride	+	179.1	150.0 (23)	15.2
	(N,N-二乙基甲苯-2,5-二胺盐酸盐)			135.1 (36)	
16	4-amino-3-nitrophenol	-	153.2	123.0 (14)	14.1
	(4-氨基-3-硝基苯酚)			122.0 (25)	
17	m-phenylenediamine	+	109.2	65.2 (31)	4.6
	(m-苯二胺)			92.1 (22)	
18	2,4-diaminophenoxy ethanol hydrochloride	+	169.1	124.1 (25)	6.2
	(2,4-二氨基苯氧基乙醇盐酸盐)			108.0 (27)	
19	hydroquinone	-	109.0	108.0 (21)	5.2
	(氢醌)			81.0 (16)	
20	4-amino-m-cresol	+	124.0	109.2 (31)	7.4
	(4-氨基-m-甲酚)			77.2 (35)	
21	2-amino-3-hydroxypyridine	+	111.1	94.0 (25)	2.7
	(2-氨基-3-羟基吡啶)			66.2 (39)	
22	N,N-bis(2-hydroxyethyl)-p-phenylenediamine sulfate	+	197.1	121.1 (36)	9.4
	(N,N-双(2-羟乙基)-p-苯二胺硫酸盐)			152.2 (22)	
23	p-methylaminophenol sulfate	+	124.0	77.1 (43)	9.9
	(p-甲基氨基苯酚硫酸盐)			109.1 (31)	
24	4-nitro-o-phenylenediamine	+	154.0	137.0 (19)	13.6
	(4-硝基-o-苯二胺)			107.0 (28)	
Dye No.	Dye	Ionization mode	Parent ion (m/z)	Daughter ion (m/z) (collision energy/eV)	t_R_/min
25	2,6-diaminopyridine	+	110.0	66.1 (33)	4.6
	(2,6-二氨基吡啶)			93.0 (30)	
26	N,N-diethyl-p-phenylenediamine sulfate	+	165.1	121.0 (21)	14.3
	(N,N-二乙基-p-苯二胺硫酸盐)			136.2 (28)	
27	6-hydroxyindole	+	134.0	107.1 (28)	15.7
	(6-羟基吲哚)			77.1 (42)	
28	4-chlororesorcinol	-	143.2	79.0 (19)	15.3
	(4-氯雷琐辛)			107.0 (16)	
29	2,7-naphthalenediol	-	159.1	130.0 (33)	15.9
	(2,7-萘二酚)			102.2 (33)	
30	N-phenyl-p-phenylenediamine	+	185.1	93.1 (29)	17.4
	(N-苯基-p-苯二胺)			108.1 (31)	
31	1,5-naphthalenediol	-	159.1	115.1 (25)	15.8
	(1,5-萘二酚)			131.0 (27)	
32	1-naphthol	-	143.1	115.1 (34)	17.8
	(1-萘酚)			41.0 (49)	

## 2 结果与讨论

### 2.1 分组的确定

基于文献^[13⇓-15]^数据、91批注册样品的标识配方及1462批监督抽检样品的检测结果,总结了染发类产品中32种染料的使用情况,其中常用染料17种(平均使用频率>1%),较少用染料7种(平均使用频率<1%),禁用染料8种。在《规范》中,17种常用染料分3个HPLC体系测定,标准溶液分3组配制避免相互干扰,但实际样品测定时,样品中所含有的处于不同分组的染料相互间往往存在干扰。本方法将32种染料分为两组,分别在两个HPLC条件下检测:将17种常用染料及3种较少用染料分在Ⅰ组,使用HPLC条件1分离;8种禁用染料及4种较少用染料分在Ⅱ组,使用HPLC条件2分离。32种染料的使用频率及分组见表3。

**表 3:** 32种染料的使用频率及分组

Dye No.	Frequency of dye use/%					Group	Dye No.	Frequency of dye use/%					Group
	Register samples	Supervise sampling samples	Ref. [13]	Ref. [14]	Ref. [15]			Register samples	Supervise sampling samples	Ref. [13]	Ref. [14]	Ref. [15]	
1	37.4	71.1	37.4	78.3	59.8	Ⅰ	17	-	0.3	-	-	-	Ⅱ
2	54.9	36.5	33.0	64.0	59.8	Ⅰ	18	18.7	32.2	28.6	3.9	13.4	Ⅰ
3	29.7	25.0	32.0	44.8	36.6	Ⅰ	19	-	-	-	-	-	Ⅱ
4	45.1	70.9	39.9	54.2	56.1	Ⅰ	20	3.3	5.0	12.3	5.4	8.5	Ⅰ
5	-	-	-	-	-	Ⅱ	21	-	7.0	10.3	-	7.3	Ⅰ
6	5.5	0.3	-	-	-	Ⅰ	22	8.8	10.2	14.3	-	8.5	Ⅰ
7	-	4.4	-	10.8	2.4	Ⅱ	23	-	1.0	-	-	1.2	Ⅱ
8	45.1	77.3	52.2	84.2	52.4	Ⅰ	24	-	0.2	-	4.4	1.2	Ⅱ
9	-	0.1	-	-	-	Ⅰ	25	-	1.0	-	-	-	Ⅰ
10	-	0.1	-	-	-	Ⅱ	26	-	0.1	-	-	-	Ⅱ
11	59.3	29.5	43.3	25.1	42.7	Ⅰ	27	-	1.5	-	-	-	Ⅰ
12	24.2	8.5	19.7	6.4	8.5	Ⅰ	28	-	6.8	-	-	2.4	Ⅰ
13	3.3	0.1	-	-	1.2	Ⅱ	29	-	0.1	-	-	-	Ⅱ
14	27.5	19.2	10.8	-	1.2	Ⅰ	30	-	-	-	-	-	Ⅱ
15	-	0.1	-	-	-	Ⅱ	31	-	3.6	-	-	-	Ⅰ
16	3.3	0.1	-	-	-	Ⅰ	32	15.4	10.5	10.8	3.9	6.1	Ⅰ

-: no usage is displayed.

### 2.2 干扰研究

#### 2.2.1 32种染料间的干扰情况

32种染料在两个HPLC体系下的分离图谱如图1、图2所示。在HPLC条件1下,有10个组分未完全分离,分别为2,6-二氨基吡啶和间苯二胺、*o*-氨基苯酚和间苯二酚、*p*-甲基氨基苯酚硫酸盐和*N*,*N*-双(2-羟乙基)-*p*-苯二胺硫酸盐、甲苯-3,4-二胺和4-硝基-*o*-苯二胺、1,5-萘二酚和2,7-萘二酚。其中,除*o*-氨基苯酚和间苯二酚需要用HPLC-MS/MS确证外,其余8个组分均可在HPLC条件2中完全分离,这两个组分中*o*-氨基苯酚在2015年被列为禁用染料,因此,本方法的分组条件能够避免32种染料组分间的相互干扰。

**图 1:**
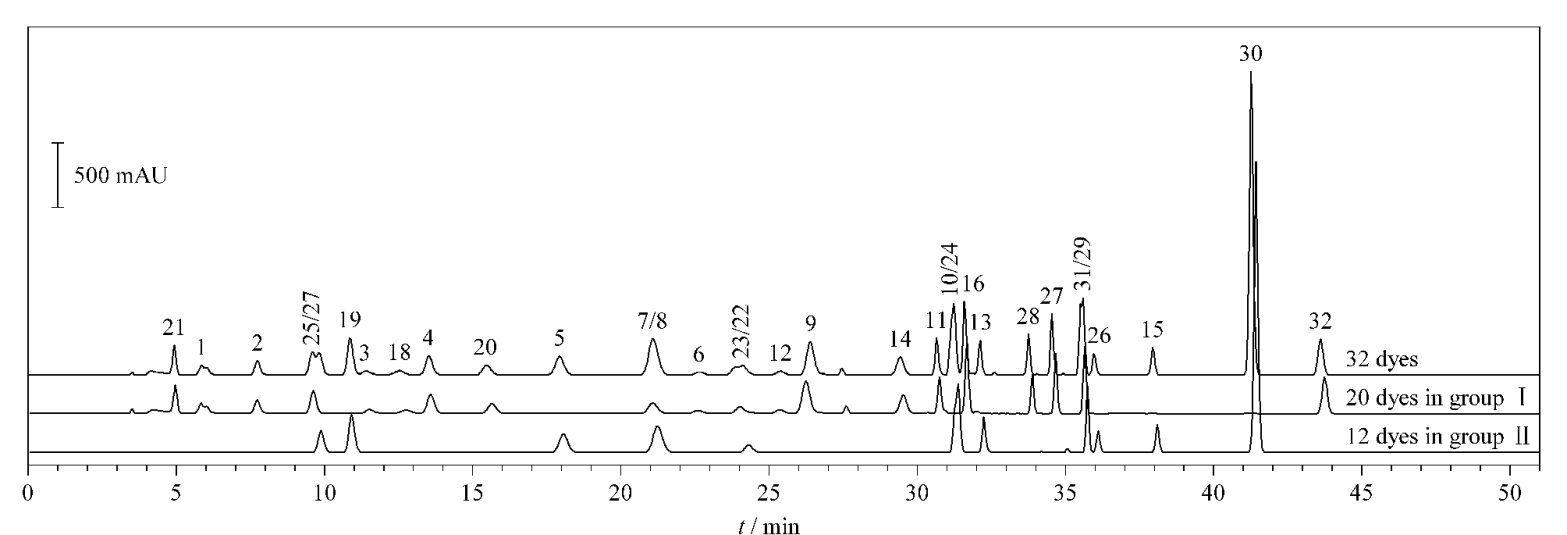
HPLC条件1下32种染料的色谱图

**图 2:**
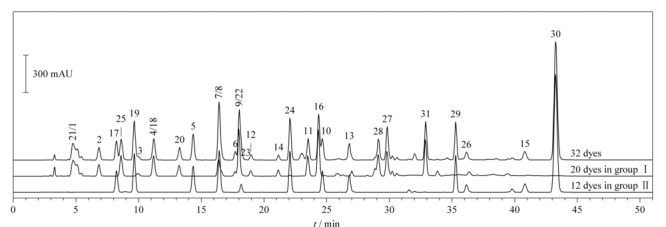
HPLC条件2下32种染料的色谱图

#### 2.2.2 其他常用染料的干扰情况

方法开发时还应尽量避免32种以外可能使用的染料对样品检测的干扰。本文试验了32种以外的其他15种常用染料对被测组分的干扰情况,保留时间(*t*_R_)及干扰结果如表4所示,2-氨基-4-羟乙氨基茴香醚和羟苯并吗啉对苯基甲基吡唑啉酮的检测在两个HPLC条件下存在干扰,其余组分均无干扰或可用其中一个色谱条件排除。

**表 4:** 其他15种常用染料的HPLC保留时间及可能干扰的32种染料的序号

Compound	t_R_/min			Disturbed dye Nos.	
	Condition 1	Condition 2		Condition 1	Condition 2
2-Amino-4-hydroxyethyl aminoanisyl ether (2-氨基-4-羟乙氨基茴香醚)	29.58	22.18		No. 14	No. 14
Hydroxybenzimoline (羟苯并吗啉)	29.58	21.30		No. 14	No. 14
2,6-Dihydroxyethyl aminotoluene (2,6-二羟乙基氨甲苯)	31.31	27.28		No. 24; No. 16	No. 13
3-Nitro-p-hydroxyacetaminophen (3-硝基对羟乙氨基酚)	32.43	27.95		No. 10	/
2-Methyl-5-hydroxyethylaminophenol (2-甲基-5-羟乙氨基苯酚)	32.65	28.81		/	No. 28
Hydroxyethyl-3,4-methylenedioxyaniline hydrochloride	33.53	30.25		No. 28	No. 27
(羟乙基-3,4-亚甲二氧基苯胺盐酸盐)					
4-Hydroxyalanine-3-nitrophenol (4-羟丙氨基-3-硝基苯酚)	33.88	32.24		No. 28	No. 31
HC yellow No. 4 (HC黄4号)	34.792	34.29		No. 27; No. 13	/
5-Amino-6-chloro-o-cresol (5-氨基-6-氯-邻甲酚)	35.22	30.25		No. 31; No. 29	No. 27
HC yellow No. 2 (HC黄2号)	37.21	37.95		/	/
5-Amino-4-chloro-o-cresol (5-氨基-4-氯-邻甲酚)	37.96	39.26		/	/
Hydroxyethyl-2-nitro-p-toluidine (羟乙基-2-硝基对甲苯胺)	39.87	45.00		/	/
Disperse violet No. 1 (分散紫1号)	41.67	56.60		No. 30	/
HC orange No. 1 (HC橙1号)	48.67	59.60		/	/
HC red No. 1 (HC红1号)	49.47	59.07		/	/

/: no interference.

### 2.3 色谱条件优化

#### 2.3.1 色谱柱的选择

《规范》采用酰胺类C_16_柱,适合分离苯胺类染料,但通用性欠佳且离子对试剂对色谱柱损伤大。本文选择杂化、包膜或聚合物封端技术的C_18_柱,可屏蔽硅羟基及金属离子影响,通过用磷酸盐改善苯胺类峰形,尝试了5 μm粒径柱及2.7 μm粒径核壳柱,两种色谱柱均可满足分离要求。核壳C_18_柱分离时间短、效率高,但出峰密集且粒径小易堵塞,考虑大规模监督抽检使用及实际样品中可能存在的杂质干扰,故选择5 μm粒径C_18_柱。市售3种具有杂化或者封端技术的5 μm粒径C_18_柱(规格为250 mm×4.6 mm, 5 μm)在HPLC条件1下Ⅰ组20种染料的分离结果如图3所示,方法通用性及分离效果均能满足要求。

**图 3:**
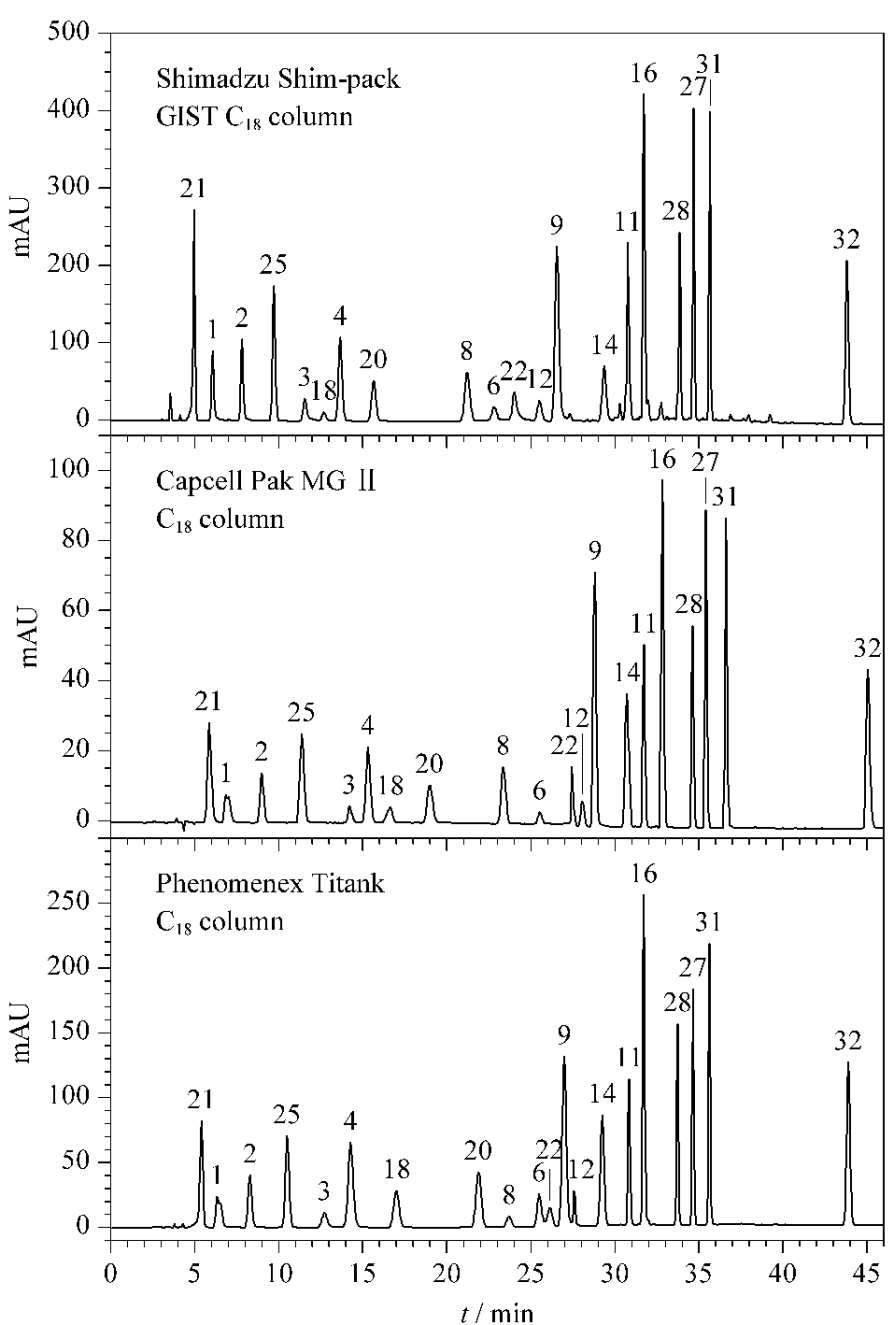
采用不同品牌色谱柱时HPLC条件1下Ⅰ组染料的色谱图

#### 2.3.2 流动相的选择

以Ⅰ组染料成分的分离效果作为流动相种类、pH值和柱温选择的判定标准。在有机相的选择上,甲醇和乙腈选择性各异,对相邻组分的分离效果不同,单独使用甲醇或乙腈均不能完全分离:使用甲醇时,组分出峰时间后移,分离效果差;使用乙腈时,*N*,*N*-双(2-羟乙基)-*p*-苯二胺硫酸盐和2-甲基雷琐辛不能完全分离,同时使用甲醇和乙腈时分离效果可满足要求。对于磷酸盐缓冲液,经试验,钾盐分离效果较钠盐、铵盐好,尤其对间苯二酚、2-氯-*p*-苯二胺硫酸盐、*N*,*N*-双(2-羟乙基)-*p*-苯二胺硫酸盐和2-甲基雷琐辛组分的分离。两种钾盐比较,使用磷酸氢二钾时,2-氯-*p*-苯二胺硫酸盐和间苯二酚峰形更佳。采用不同流动相种类时HPLC条件1下Ⅰ组20种染料的分离结果见图4。综上,使用三元梯度分析,选择磷酸氢二钾-甲醇-乙腈作为流动相。

**图 4:**
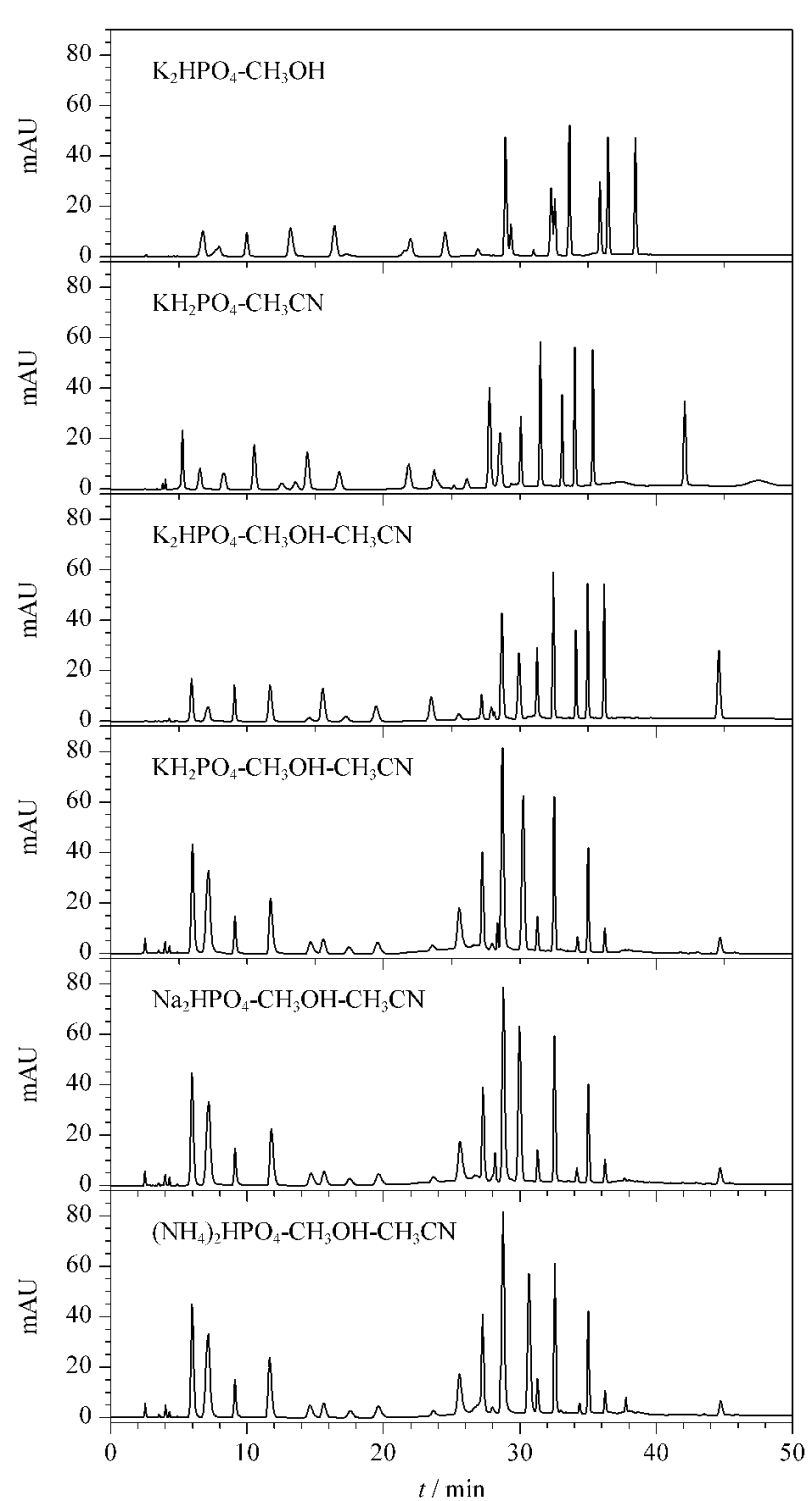
采用不同流动相种类时HPLC条件1下Ⅰ组染料的色谱图

#### 2.3.3 pH的选择

32种染料覆盖酸/碱性物质,降低流动相pH值有利于防止酸性物质离子化,使其以分子形式存在于色谱柱上得到分离,而提高pH值则相反,故需确定能同时满足两性物质检测的pH条件。未使用离子对,流动相的酸度变化显著影响出峰时间,碱性条件更有利于分离,尤其是2-硝基-*p*-苯二胺和苯基甲基吡唑啉酮、4-氨基-2-羟基甲苯和4-氨基-3-硝基苯酚、4-氯雷琐辛和6-羟基吲哚的分离。经试验,HPLC条件1下不同流动相pH条件时Ⅰ组20种染料的分离结果见图5。pH为7.5时能实现20种组分的完全分离,故选择流动相pH为7.5。

**图 5:**
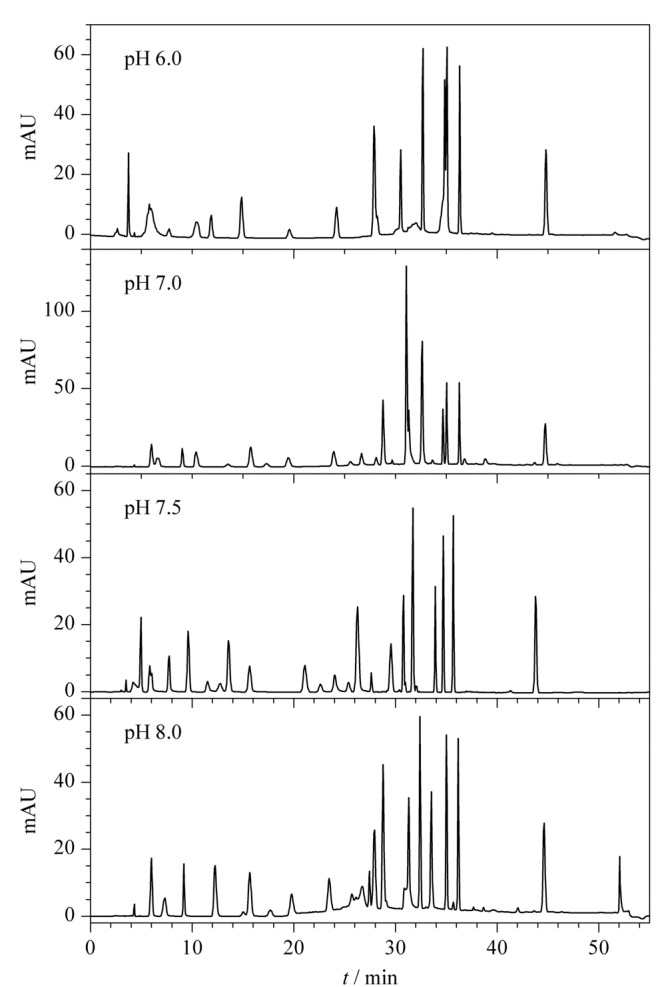
采用不同流动相pH时HPLC条件1下Ⅰ组染料的色谱图

#### 2.3.4 柱温的选择

考察了20、25、30、40 ℃下Ⅰ组20种染料在HPLC条件1下的分离情况,温度过高会影响色谱柱的使用寿命,且随着温度升高,各染料组分保留时间逐渐缩短,分离度下降,20和25 ℃分离效果较好,综合考虑温度控制的难易程度和组分的分离度,选择色谱柱温度为25 ℃。

### 2.4 样品前处理条件优化

#### 2.4.1 抗氧化剂种类及使用量的选择

市售染发类产品中抗氧化剂主要有亚硫酸钠、亚硫酸氢钠、抗坏血酸和异抗坏血酸。本研究以自制不含抗氧化剂、染料及其中间体的样品(主要成分为水、鲸蜡硬脂醇、矿油、平平加、西曲氯胺、聚二甲基硅氧烷、EDTA二钠)作为空白样品,称取0.5 g于25 mL比色管中,加入500 mg/L的Ⅰ组20种染料的储备溶液1.0 mL,以此作为加标样品考察了上述4种抗氧化剂及亚硫酸氢钠-抗坏血酸混合使用对Ⅰ组20种染料的抗氧化能力,结果表明,5种抗氧化溶液在48 h内抗氧化能力相当,抗坏血酸和异抗坏血酸溶剂峰较大,亚硫酸氢钠溶剂峰最小,因此确定为样品前处理的抗氧化剂。

在加标样品中分别加入10 g/L亚硫酸氢钠溶液0、0.5、1.0、2.0、5.0、10.0 mL, 48 h稳定性数据表明,在不添加抗氧化剂的条件下,2,4-二氨基苯氧基乙醇盐酸盐、2-氯-*p*-苯二胺硫酸盐、*N*,*N*-双(2-羟乙基)-*p*-苯二胺硫酸盐、2-甲基雷琐辛4种组分在第二天检测时降解明显,回收率下降。亚硫酸氢钠添加量在1.0 mL以上时结果稳定,为了保证不同样品基质下抗氧化能力的稳定性,采用亚硫酸氢钠添加量为2.0 mL。

#### 2.4.2 提取溶剂的选择

试验了无水乙醇-水(1∶1, v/v)溶液、甲醇-水(1∶1, v/v)溶液、乙腈-水(1∶1, v/v)溶液3种提取溶剂对Ⅰ组染料的提取效果。3种提取溶剂均能取得理想的回收率,结果无显著差异,考虑溶剂的安全性,采用无水乙醇-水(1∶1, v/v)溶液作为样品处理的提取溶剂。

### 2.5 HPLC-MS/MS条件的选择

HPLC采用保留时间和光谱吸收定性,具有非唯一性,在实际样品检测中,如出现相同保留时间下光谱吸收匹配存疑、杂质峰干扰或检出结果与标签、注册资料不符等情况,可按照未知物确认程序采用HPLC-MS/MS方法进行确证。

分别配制32种染料溶液(氢醌质量浓度为10 mg/L,其他组分质量浓度为1 mg/L),以甲醇-水(1∶1, v/v)为基准流动相,采用直接进样方式将标准溶液注入离子源,对32种染料的MRM条件进行优化,母离子*m/z*、子离子*m/z*、碰撞能和保留时间见表2。

分别选用5 mmol/L乙酸-乙腈、5 mmol/L乙酸铵-乙腈、5 mmol/L甲酸-乙腈、水-乙腈、0.1%氨水-乙腈等优化流动相组成。经实验验证,甲酸-乙腈条件下峰形欠佳;水-乙腈和氨水-乙腈条件下,负离子模式检测的组分拖尾严重。乙酸-乙腈条件下质谱响应最大、峰形最好,但在正离子模式下,部分组分的色谱峰基本不保留,分离困难。乙酸铵-乙腈为流动相时,各组分的色谱保留和分离度较好,但负离子模式下响应低,尤其是间苯二酚、2-甲基雷琐辛、4-氨基-3-硝基苯酚和氢醌等组分。综合质谱响应和质谱保留,选用乙酸铵-乙腈为正离子模式下的流动相;乙酸-乙腈为负离子模式下的流动相。

比较了不同浓度的乙酸和乙酸铵(5、10、20 mmol/L)的效果,结果表明,提高乙酸和乙酸铵的浓度,组分的分离和响应没有显著影响,过高浓度的酸和盐损害色谱柱的使用寿命,因此,选择乙酸和乙酸铵的浓度为5 mmol/L。

32种染料中包含的6对同分异构体分别是*p*-苯二胺、*o*-苯二胺和*m*-苯二胺;*p*-氨基苯酚、*m*-氨基苯酚和*o*-氨基苯酚;甲苯-2,5-二胺硫酸盐和甲苯-3,4-二胺;4-氨基-2-羟基甲苯、6-氨基-*m*-甲酚、4-氨基-*m*-甲酚和*p*-甲基氨基苯酚硫酸盐;间苯二酚和氢醌;2,7-萘二酚和1,5-萘二酚。在上述HPLC条件下,各同分异构体组分的保留时间如表2所示,6对同分异构体均完全分离。

### 2.6 方法学考察

#### 2.6.1 标准曲线及检出浓度

在本方法条件下考察一定浓度范围内HPLC和HPLC-MS/MS法测得的各被测物质峰面积对应浓度的线性相关性。在信噪比为3、样品称样量0.5 g、稀释25倍条件下计算本方法的检出限。线性回归方程、相关系数(*r*^2^)、检出限见表5。

**表 5:** HPLC和HPLC-MS/MS方法中32种染料的线性范围、相关系数及检出限

Dye No.	HPLC				HPLC-MS/MS		
	Linear range/(mg/L)	r^2^	LOD/(μg/g)		Linear range/(mg/L)	r^2^	LOD/(μg/g)
1	10.0-499.5	0.9999	40.0		0.1-4.2	0.9986	0.04
2	10.0-499.0	0.9999	20.0		0.1-4.3	0.9986	0.04
3	10.0-500.0	0.9998	40.0		0.1-4.2	0.9999	0.2
4	10.0-501.2	0.9979	20.0		0.1-4.2	0.9950	0.2
5	9.9-495.0	1.0000	19.8		0.1-4.2	0.9999	0.02
6	9.9-497.1	0.9996	39.9		0.1-4.3	0.9995	0.04
7	10.2-509.6	1.0000	10.2		0.1-4.1	0.9962	0.02
8	10.0-501.6	1.0000	20.0		0.1-4.0	0.9991	0.4
9	10.0-500.0	1.0000	10.0		0.1-4.4	0.9991	0.02
10	10.0-501.0	1.0000	10.0		0.1-4.3	0.9994	0.02
Dye No.	HPLC				HPLC-MS/MS		
	Linear range/(mg/L)	r^2^	LOD/(μg/g)		Linear range/(mg/L)	r^2^	LOD/(μg/g)
11	10.0-500.0	0.9979	19.7		0.1-4.1	0.9999	0.02
12	10.0-500.0	0.9999	40.0		0.1-4.2	0.9992	0.4
13	10.0-490.0	1.0000	19.6		0.1-3.8	0.9984	0.02
14	10.0-500.2	0.9991	19.6		0.1-4.2	0.9996	0.02
15	10.0-501.1	1.0000	40.1		0.1-4.2	0.9990	0.01
16	10.0-498.7	1.0000	20.1		0.1-4.2	0.9995	0.04
17	10.0-500.9	0.9999	10.0		0.1-3.7	0.9981	0.02
18	9.8-489.6	0.9974	39.2		0.1-4.0	0.9999	0.04
19	10.0-500.0	0.9997	10.0		2.0-79.7	0.9979	8.0
20	9.9-493.1	0.9995	19.9		0.1-4.0	0.9901	0.04
21	9.9-496.9	1.0000	39.8		0.1-4.2	0.9948	0.04
22	9.7-486.8	0.9999	39.6		0.1-4.2	0.9974	0.4
23	9.9-493.9	0.9999	39.5		0.1-4.5	0.9937	0.02
24	9.9-493.4	1.0000	9.9		0.1-3.8	0.9994	0.02
25	9.9-494.9	1.0000	19.8		0.1-4.3	0.9993	0.02
26	9.9-496.6	1.0000	39.7		0.1-4.0	0.9990	0.2
27	9.8-491.0	1.0000	9.7		0.1-4.3	0.9988	0.2
28	10.0-501.0	1.0000	9.9		0.1-4.3	0.9999	0.2
29	10.1-503.1	1.0000	10.1		0.1-4.4	0.9999	0.04
30	9.9-497.4	1.0000	9.9		0.1-4.6	0.9965	0.01
31	10.1-505.1	1.0000	10.0		0.1-3.8	0.9999	0.2
32	10.0-498.3	1.0000	10.0		0.1-3.8	0.9980	0.2

结果表明,HPLC方法中32种染料在大约10~500 mg/L范围内线性相关性良好(*r*^2^>0.99),检出限为9.7~40.1 μg/g; HPLC-MS/MS方法中氢醌的线性范围为2.0~79.7 mg/L,检出限为8.0 μg/g,其他组分的线性范围约为0.1~4 mg/L,检出限为0.01~0.4 μg/g。

#### 2.6.2 精密度及稳定性

分别以10、500 mg/L和0.1、4 mg/L的混合标准溶液考察HPLC和HPLC-MS/MS法的精密度和标准溶液的稳定性。每个浓度连续进样6次,计算日内精密度的RSD,3天内每天连续进样6次,计算日间精密度的RSD;于0、2、4、8、12、24、48、72 h进样,计算48 h和72 h内稳定性的RSD。日内、日间精密度和稳定性结果见表6。

**表 6:** HPLC和HPLC-MS/MS方法中32种染料的精密度(*n*=6)和稳定性

Dye No.	HPLC (10 mg/L)							HPLC (500 mg/L)						HPLC-MS/MS (0.1 mg/L)						HPLC-MS/MS (4 mg/L)						
	Precision (n=6)			Stability				Precision (n=6)			Stability			Precision (n=6)			Stability			Precision (n=6)			Stability			
	1 d	3 d		48 h	72 h			1 d	3 d		48 h	72 h		1 d	3 d		48 h	72 h		1 d	3 d		48 h	72 h		
1	2.0	4.6		3.9	4.5			3.8	2.4		1.6	1.8		3.2	3.3		3.2	4.5		5.6	4.8		5.2	4.5		
2	3.2	3.9		4.1	4.2			2.1	3.1		2.3	2.3		6.4	7.7		2.5	3.7		8.1	9.3		3.6	3.1		
3	5.0	7.5		4.0	3.8			4.8	8.0		4.9	5.5		9.3	7.4		3.9	5.5		9.8	8.7		6.1	5.1		
4	1.7	3.3		4.7	4.9			1.0	3.2		1.2	1.6		8.8	10		4.2	8.4		9.3	8.8		5.8	6.2		
5	0.7	1.2		0.8	1.0			0.1	0.3		0.2	0.4		4.1	8.1		2.0	3.7		8.1	8.1		3.7	3.2		
6	2.7	4.5		3.9	5.3			2.3	3.8		4.3	4.3		9.8	9.4		3.4	11		9.4	8.3		5.6	5.3		
7	1.2	1.8		0.9	0.9			0.1	0.3		0.1	0.3		6.5	8.0		2.6	4.9		7.6	7.2		4.2	4.3		
8	4.8	8.7		4.3	5.2			1.8	4.4		2.6	2.7		5.2	8.7		3.4	11		8.3	8.7		4.5	7.9		
9	2.5	4.2		1.0	2.6			0.2	3.4		0.9	2.8		9.2	7.6		2.4	4.6		6.1	8.5		4.6	4.6		
10	1.3	1.6		1.1	1.1			0.3	0.4		0.1	0.2		9.9	9.4		2.4	3.6		8.4	9.2		3.5	3.2		
11	2.0	5.5		4.6	4.5			0.2	4.9		0.5	1.2		4.6	4.7		2.9	4.4		5.5	5.2		4.1	3.8		
12	4.2	9.2		1.9	2.8			0.7	4.8		4.0	3.8		9.3	9.2		3.0	6.5		9.3	9.6		3.1	4.0		
13	0.7	1.5		1.5	1.8			0.1	0.6		0.3	0.42		8.4	7.7		1.3	2.2		9.3	9.5		1.6	1.4		
14	1.0	6.6		1.1	3.5			0.7	9.0		3.3	5.2		2.4	8.7		2.0	8.3		3.7	8.7		1.8	5.9		
Dye No.	HPLC (10 mg/L)						HPLC (500 mg/L)							HPLC-MS/MS (0.1 mg/L)						HPLC-MS/MS (4 mg/L)							
	Precision (n=6)			Stability			Precision (n=6)				Stability			Precision (n=6)			Stability			Precision (n=6)			Stability				
	1 d	3 d		48 h	72 h		1 d		3 d		48 h	72 h		1 d	3 d		48 h	72 h		1 d	3 d		48 h		72 h		
15	2.8	3.3		4.8	4.5		0.2		0.5		0.2	0.2		8.8	8.7		1.1	4.6		9.1	9.2		3.4		3.8		
16	0.8	0.9		0.7	0.8		0.4		4.3		1.7	3.3		6.2	4.9		1.4	9.5		6.5	6.2		6.0		7.6		
17	0.7	1.0		1.1	1.0		0.1		1.2		1.1	1.3		7.0	5.6		2.5	4.1		6.8	6.2		3.5		3.0		
18	4.5	7.3		4.3	7.4		4.7		7.8		5.0	6.1		9.5	9.9		1.8	7.4		5.5	10		3.1		9.8		
19	4.8	10		4.1	15.5		0.1		0.2		0.1	0.1		5.2	6.3		3.9	5.9		6.9	7.5		4.9		5.8		
20	3.3	9.0		4.1	5.4		2.7		5.0		3.1	3.3		8.6	8.5		1.6	3.5		8.8	9.2		2.6		2.3		
21	2.6	2.5		4.5	4.2		1.1		1.0		0.8	0.8		8.8	8.1		2.0	4.8		8.0	6.9		4.0		3.7		
22	4.4	5.2		4.2	4.0		0.2		6.8		1.0	3.8		7.2	11		3.2	6.9		9.8	13		4.0		8.3		
23	2.1	9.8		3.5	14		0.5		2.1		1.3	1.8		9.3	7.9		1.3	4.5		7.5	8.8		4.9		4.2		
24	0.4	0.5		0.6	0.6		0.1		0.3		0.1	0.2		6.2	6.9		1.9	7.0		8.5	7.2		1.5		4.4		
25	1.5	6.1		4.4	4.3		0.4		3.5		0.5	2.5		6.1	4.3		1.7	3.4		5.8	5.5		2.6		2.8		
26	2.6	3.7		1.9	1.8		0.1		0.2		0.2	0.2		8.3	6.0		2.8	4.0		4.4	4.4		3.7		3.5		
27	1.4	0.8		1.0	1.0		0.2		0.9		0.7	1.0		8.6	8.9		2.5	5.5		8	8.2		2.2		4.9		
28	2.5	4.6		4.5	4.2		0.1		1.0		0.8	1.0		8.7	7.6		1.4	6.8		9.7	8.5		1.9		5.4		
29	0.6	0.6		0.8	0.7		0.1		0.3		0.1	0.2		5.1	7.3		2.2	3.1		5.5	7.5		4.6		4.4		
30	0.5	0.6		1.2	1.1		0.1		0.4		0.1	0.3		8.6	6.1		2.5	3.3		8.8	7.6		3.1		2.9		
31	1.0	1.2		1.6	1.7		0.1		0.5		0.2	0.4		7.5	6.4		2.7	5.8		8.1	7.8		2.2		4.5		
32	1.7	1.6		3.7	3.5		0.1		0.9		0.2	0.4		6.0	9.9		2.0	6.7		9.1	9.4		3.1		5.6		

结果表明,32种染料的精密度和48 h稳定性的RSD在HPLC方法中小于5%, HPLC-MS/MS方法中小于10%,符合两个检测方法的精密度和稳定性要求。部分组分72 h的稳定性RSD>10%,建议配制染料组分的单标储备溶液保存,混合标准系列溶液在48 h内使用。

#### 2.6.3 加标回收率

取空白样品0.5 g(精确到0.001 g)于25 mL具塞比色管中,分别精密加入500 mg/L混合标准系列溶液和5 g/L混合标准储备溶液1.0 mL,作为加标量分别为1000 μg/g和10000 μg/g的HPLC方法加标回收溶液;精密加入氢醌(500 mg/L)和其余组分混合标准溶液(25 mg/L)0.5 mL,作为加标量分别为25 μg/g的HPLC-MS/MS方法的加标回收溶液。平行制备6份,HPLC和HPLC-MS/MS法的回收率及RSD结果如表7所示,两个方法的回收率范围分别为85.1%~114.7%(RSD<5%)和82.6%~114.9%(RSD<10%)。

**表 7:** HPLC和HPLC-MS/MS方法中32种染料的加标回收率(*n*=6)

Dye No.	HPLC^1)^			HPLC^2)^			HPLC-MS/MS^3)^		Dye No.	HPLC^1)^			HPLC^2)^			HPLC-MS/MS^3)^		
	Recovery/ %	RSD/ %		Recovery/ %	RSD/ %		Recovery/ %	RSD/ %		Recovery/ %	RSD/ %		Recovery/ %	RSD/ %		Recovery/ %		RSD/ %
1	86.0	1.6		93.8	2.3		114.0	2.9	17	103.5	0.3		97.7	0.5		114.0		1.1
2	104.7	1.1		96.8	1.1		114.2	3.9	18	85.1	2.6		85.3	4.1		111.0		3.4
3	85.3	1.4		85.2	5.2		108.9	3.8	19	105.3	0.8		87.6	0.5		98.9		6.0
4	106.2	1.7		99.8	1.4		111.8	3.0	20	114.7	2.0		97.3	4.2		103.3		5.3
5	103.9	1.2		98.6	0.5		111.9	2.6	21	99.9	1.3		95.1	1.0		105.3		4.3
6	87.9	1.2		92.7	1.8		105.6	1.7	22	100.3	2.9		87.2	4.5		104.4		8.4
7	100.6	0.6		95.2	0.5		105.6	1.4	23	93.6	2.5		86.8	0.7		95.8		4.8
8	98.2	1.4		99.7	5.7		107.9	2.6	24	98.1	1.1		95.4	4.2		114.5		2.4
9	97.9	1.3		94.6	1.2		106.6	1.7	25	90.9	1.3		90.2	2.4		114.9		1.4
10	100.4	1.1		99.2	0.5		114.4	1.5	26	101.6	2.3		99.6	0.7		106.0		7.1
11	89.1	1.6		109.4	3.4		100.0	1.2	27	89.9	1.8		95.0	1.8		95.8		2.0
12	85.1	0.6		103.1	4.0		103.3	3.3	28	97.0	1.9		95.9	1.0		98.4		2.1
13	98.1	1.3		98.7	0.4		96.5	1.4	29	93.6	3.5		97.1	1.9		106.4		5.5
14	85.6	2.2		93.2	1.2		112.7	3.8	30	97.6	1.2		98.4	0.5		102.2		3.8
15	101.2	2.8		98.3	0.4		95.7	5.4	31	89.8	3.9		86.5	3.8		96.6		9.8
16	92.7	2.1		97.8	1.1		93.5	5.8	32	93.3	2.6		91.0	2.1		82.6		5.3

Spiked levels: 1) 1000 μg/g; 2) 10000 μg/g; 3) 25 μg/g.

### 2.7 样品测定

为比较3个方法对样品检测结果的一致性,本实验收集10个市售氧化型染发产品,分别采用《规范》“7.2对苯二胺等32种组分”、本文的HPLC和HPLC-MS/MS方法进行检测。其中1个染发产品的HPLC条件1下的色谱图见图6。10批样品共检出16种染料,分别为*p*-苯二胺、*p*-氨基苯酚、甲苯-2,5-二胺硫酸盐、*m*-氨基苯酚、间苯二酚、4-氨基-2-羟基甲苯、2-甲基雷琐辛、苯基甲基吡唑啉酮、2,4-二氨基苯氧基乙醇盐酸盐、4-氨基-*m*-甲酚、2-氨基-3-羟基吡啶、*N*,*N*-双(2-羟乙基)-*p*-苯二胺硫酸盐、2,6-二氨基吡啶、6-羟基吲哚、4-氯雷琐辛、1-萘酚。检出含量范围为58~25160 μg/g, 3个方法检测结果的RSD为1.9%~10.1%。所有检出组分均包含在本方法的Ⅰ组中。

**图 6:**
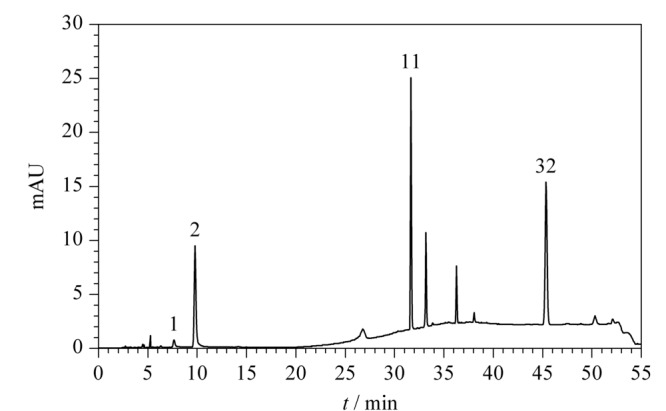
1个染发产品在HPLC条件1下的色谱图

## 3 结论

本文建立了32种氧化型染料的HPLC检测方法和HPLC-MS/MS确证方法,相比《规范》标准方法,本方法使用常规C_18_柱替换酰胺类C_16_柱,不使用离子对试剂,增加了方法的通用性;基于染料使用频率分组分离的HPLC方法能够避免实际样品中染料组分间的干扰,常用染料可在HPLC条件1中完成检测,减少了注册检验中染料组分检测的工作量;与文献^[10⇓-12]^报道方法相比,本方法能够全部覆盖目前监督抽检要求的染料组分,并建立了HPLC-MS/MS确证方法,更好地满足监督检验的需求。方法简便准确,通用性和稳定性良好,能够作为染发类产品中多种染料检测的标准推广方法。
